# Successful management of unsuspected retroperitoneal paraganglioma via the use of combined epidural and general anesthesia: a case report

**DOI:** 10.1186/1752-1947-7-58

**Published:** 2013-02-28

**Authors:** Katarina Tomulic, Jadranka Pavicic Saric, Branislav Kocman, Anita Skrtic, Natasa Viskovic Filipcic, Ivana Acan

**Affiliations:** 1 Department of Anesthesiology and Intensive Care, University Hospital Merkur, Zajceva 19, Zagreb, Croatia; 2 Department of Surgery, University Hospital Merkur, University of Zagreb, School of Medicine, Zajceva 19, Zagreb, Croatia; 3 Department of Pathology, University Hospital Merkur, University of Zagreb, School of Medicine, Zajceva 19, Zagreb, Croatia

**Keywords:** Epidural analgesia, Invasive hemodynamic monitoring, Paraganglioma/pheochromocytoma, Therapeutic protocols

## Abstract

**Introduction:**

Similar to pheochromocytomas, paragangliomas can secrete catecholamines, although they are usually non-functional and clinical presentation is non-specific. We present a case of accidental, intra-operatively diagnosed neuroendocrine-active sympathetic paraganglioma, which was suspected and confirmed during elective retroperitoneal tumor removal.

**Case presentation:**

A 25-year-old Caucasian Croatian man, American Society of Anesthesiologists status 1, underwent elective surgery for retroperitoneal tumor removal. The tumor had been discovered by chance during a routine examination and was suspected to be a sarcoma. Our patient had no history of previous medical conditions nor did he have symptoms characteristic of a neuroendocrine secreting tumor. The results of ultrasound and magnetic resonance imaging studies showed a large, well demarcated retroperitoneal tumor mass in his upper abdomen localized between the aorta and vena cava, measuring approximately 9×6×4.5cm. In the operating room an epidural catheter was inserted at the T7 to T8 level prior to induction of general anesthesia. Epidural analgesia was maintained by an infusion pump with local anesthetic and opiate mixture. During the surgical excision of the tumor, hemodynamic changes occurred, with hypertension (205/110mmHg) and tachycardia (up to 120 beats/minute). In spite of the fact that the surgical field of work did not include adrenal glands whose direct manipulation could explain this occurrence, there was a high degree of suspicion for the presence of a neurosecreting tumor. His clinical symptoms were relieved after administration of urapidil, esmolol and magnesium sulfate. After tumor excision, our patient developed severe hypotension. Hemodynamic stability was reinstated with aggressive volume replacement, with crystalloids and colloids, vasopressors and hydrocortisone. His post-operative course was unremarkable and on the eighth post-operative day our patient was discharged from hospital, with no consequences or symptoms on follow-up two years after surgery.

**Conclusions:**

Our patient’s case emphasizes the need to consider the presence of extra-adrenal paragangliomas in the differential diagnosis of retroperitoneal tumors, despite their rare occurrence. In our patient’s case, invasive hemodynamic monitoring during combined general anesthesia and epidural analgesia and early recognition of catechol-induced symptoms raised suspicion of the existence of a paraganglioma, and this led to an adequate therapeutic approach and favorable outcome of the surgery. Pre-operative recognition of paragangliomas could lead to better pre-operative preparation, but even high clinical suspicion in undiagnosed forms during surgery and the availability of rapid and short-acting vasodilatators, α-blockers and β-blockers might favor good outcome.

## Introduction

Paragangliomas of the retroperitoneum are neuroendocrine tumors arising from the sympathoadrenal paraganglial autonomic nervous system. Most of the tumors arise in the adrenal medulla as pheochromocytoma, also known as intra-adrenal paraganglioma, while the remaining arise in aortic-sympathetic extra-adrenal paraganglia along the paravertebral axis as extra-adrenal sympathetic paragangliomas. Paragangliomas account for 15 percent to 20 percent of pheochromocytomas and have a high incidence of malignancy (13 percent to 26 percent) [1-5]. Most tumors are hormonally active (up to 60 percent) and cause symptoms associated with the excessive secretion of catecholamines. The most common symptoms include hypertension associated with headache, sweating and palpitations. Although paragangliomas are rare, the potential for release of catecholamines presents a significant clinical hazard. In fact, great care must be exercised during surgical removal of paragangliomas. Their excision is considered extremely difficult due to their high vascularity and adherence to multiple adjacent structures, including the aorta, vena cava, and renal and mesenteric vessels. Regardless, the risk of their malignant transformation justifies aggressive management [6].

We present a case of accidental, intra-operatively diagnosed functional extra-adrenal sympathetic paraganglioma in which a high level of suspicion during a surgical procedure performed using combined epidural analgesia and general anesthesia resulted in optimal hemodynamic management during surgery.

## Case presentation

A 25-year-old Caucasian Croatian man, American Society of Anesthesiologists (ASA) status 1, underwent elective surgery for removal of a retroperitoneal tumor suspected to be a sarcoma. The tumor had been discovered accidentally during a routine examination. Our patient had no history of previous medical conditions. His family history did not suggest a hereditary disorder. He had no symptoms that could be related to a neuroendocrine-secreting tumor such as hypertension, palpitations, sweating, headache, glucose intolerance or Cushing’s appearance. His height was 165cm, weight 55kg and body mass index (BMI) was 20.2kg/m^2^. Pre-operative laboratory findings, chest X-ray and electrocardiogram (ECG) results were normal; his blood pressure measured 120/70mmHg pre-operatively. Ultrasound and magnetic resonance imaging (MRI) results showed a large, well-demarcated retroperitoneal tumor mass in his upper abdomen localized between the aorta and vena cava, measuring approximately 9×6×4.5cm, leaning on the liver and pancreas and spreading towards, but not affecting, the right adrenal gland. Our patient received midazolam 7.5mg orally 30 minutes before he was taken to the operating room. After establishing intravenous access and placing standard anesthesia monitors (non-invasive measurement of blood pressure (NIBP), ECG, SpO_2_ (pulse oximetry), and temperature), an epidural catheter was inserted in the epidural space between T7 and T8 under local anesthesia. Its correct epidural placement was tested with 60mg of lidocaine (with no additional epinephrine) to exclude the possibility of intra-vascular or intra-thecal puncture. A mixture of 0.25 percent levobupivacaine 25mg and sufentanil 10μg were administered epidurally twice as a bolus dose to adjust the block level, before the induction of general anesthesia. The epidural analgesia was tested by loss of sensation to pinprick and cold, compared to an unblocked dermatome. To eliminate surgical stress during the resection in accordance with the localization of tumor as assessed by MRI, the aiming level of epidural analgesia was adjusted to be from T5 to T12. Induction of anesthesia was made with propofol 120mg, sufentanil 10μg, following rocuronium 50mg to facilitate endotracheal intubation. Our patient received an arterial line for invasive blood measurement and a central venous catheter in the right jugular vein for central venous pressure measurement. The anesthesia was maintained with sevoflurane, in a mixture of O_2_ and air (in 40:60 ratios). Epidural analgesia was maintained continuously with an infusion pump (mixture of 0.125 percent levobupivacaine 50mg with sufentanil 10μg in 50mL of saline, infusion rate 5 to 25mL/hour) and this block completely satisfied intra-operative analgesic demands.

During the surgical excision of the tumor, severe and unexpected hemodynamic changes occurred. Minimal tumor manipulation was followed with severe hypertension measuring 205/110mmHg and tachycardia up to 120 beats/minute. Due to the fact that the surgical manipulation did not affect the adrenal glands and that epidural anesthesia was tested as sufficient for the anticipated surgery and also proven to be effective for treating surgical stimuli until this change occurred, suspicion of the presence of a neurosecreting tumor was raised.

The levels of both anesthesia and analgesia were increased in order to rule out too light a level of anesthesia and, as a consequence, pain. A bolus dose of intravenous sufentanil (25μg) was given, along with increase of the sevoflurane minimum alveolar concentration (MAC) and infusion rate of epidural analgesia (for 15 minutes), but the hypertension and tachycardia could not be suppressed by these pharmacological measures.

Also, we tried to rule out the other most common causes of such hemodynamic changes. Based on our patient’s medical history we could rule out pre-existing cardiac conditions, autonomic nerve system dysfunctions or hormonal disorders such as thyroid storm. His body temperature did not rise so we ruled out the presence of malignant hyperthermia. Basic laboratory results did not suggest any severe electrolyte disorder. All the symptoms were reflecting high and unsuppressed catecholamine activity. The clinical symptoms diminished only after additional administration of intravenous urapidil 40mg, esmolol 20mg and MgSO_4_ (magnesium sulfate) 2g.

Due to a large intra-operative blood loss because of the tumor’s extremely high vascularization our patient received high volume replacement in the form of crystalloids (7500mL), colloids (2000mL), and multiple blood transfusions (red blood cells (RBC) 1400mL and fresh frozen plasma (FFP) 1000mL). Adequate central venous pressure and diuresis were maintained during the procedure.

After excision of the tumor, our patient developed hypotension, due to the ‘downregulation’ of the receptors, with a lowest blood pressure measurement of 65/35mmHg. The epidural infusion was immediately discontinued. This condition was treated with additional large volume replacement with crystalloids, colloids, and vasopressors (norepinephrine up to 0.25μg/kg/min) in order to achieve a mean arterial pressure (MAP) over 65mmHg. Additional hemodynamic stability was eventually restored after administration of a bolus dose of hydrocortisone 100mg intravenously, which eventually led to cessation of the vasopressor infusion 45 minutes later.

His post-operative course was unremarkable. After the surgery, our patient was admitted to our Intensive Care Unit (ICU) where he remained intubated and ventilated for two hours. He was hemodynamically stable, without vasopressor support. There were no episodes of hypertension or tachycardia reported. Analgesia was supplied by epidural catheter and our patient experienced no pain. He was transferred to the ward the next day and on the eighth post-operative day he was discharged from hospital. The final pathology report confirmed the presence of an extra-adrenal sympathetic paraganglioma (EASP).

## Discussion

Extra-adrenal paragangliomas have the ability, similar to pheochromocytomas, to be hormonally active. This onset of activity can result in sudden, life-threatening cardiovascular, neurological or metabolic crisis. A few patients (13 percent) may be asymptomatic in spite of high levels of circulating catecholamines due to receptor ‘downregulation’. The asymptomatic features make it difficult for clinicians to detect the tumor at an early stage [7]. Detection is even more challenging for physicians working in institutes that do not perform routine screening tests for such malignancies. The greatest danger from this clinically silent paraganglioma is hemodynamic instability when they are being manipulated during surgery, leading to lethal complications and outcomes.

Therefore, when dealing with a patent with retroperitoneal tumor, it is crucial to consider the presence of a hormonally active paraganglioma even in an asymptomatic patient.

A wide range of diagnoses should be considered in order to plan the best treatment for the patient. Grumbach *et al*. recommend the combination of biochemical and imaging tests [8]. The biochemical diagnosis of hyper-functional paraganglioma (or adrenal pheochromocytoma) is based on excessive excretion of catecholamines and their metabolites. Determination of 24-h urinary excretion of total metanephrines and catecholamines (norepinephrine, epinephrine, and dopamine) has been widely used as a screening test panel [9]. With regard to imaging techniques, MRI is associated with the lowest false negative localization rate, and radioactive iodine-labeled metaiodobenzylguanidine (MIBG) scintigraphy is the least sensitive imaging study of all. Although lacking in sensitivity, MIBG scintigraphy is highly specific and may be the only positive imaging test result in some patients [10].

In our patient, a retroperitoneal tumor was mistaken for a sarcoma on MRI findings, in spite of MRI’s reported 91 percent sensitivity for extra-adrenal tumors [11]. Due to the lack of pre-operative recognition, our patient did not receive pharmacological pre-conditioning with α-blockers and β-blockers prior to the procedure, which led to hemodynamic instability intra-operatively. As described, we established an adequate level and quality of epidural analgesia for the anticipated surgery, which meant that pharmacological sympathectomy at the desired levels was also accomplished. The depth of anesthesia at the moment of the removal of the tumor was also sufficient so that the resulting extreme hypertensive reaction and tachycardia had to be attributed to the massive release of catecholamines in an intra-abdominal localization.

Epidural analgesia is beneficial in patients who undergo open procedures with a large incision for its anesthetic and analgesic features intra-operatively and post-operatively. It is recommended for abdominal pheochromocytoma (and other neurosecreting tumor) resection in order to avoid the marked fluctuation of blood pressure that may accompany the induction of general anesthesia, and it also reduces the need for vasoactive drugs in the anesthesia management due to its sympathomimetic features [12,13]. It is an effective and safe method to prevent fluctuations in hormone levels. This combination of epidural analgesia and general anesthesia may not prevent hemodynamic fluctuations during tumor manipulation, but appears to facilitate a rapid and stable post-operative recovery [14].

Our patient’s hypertension was treated with urapidil intra-operatively and esmolol was given to suppress tachycardia. Additional hemodynamic control was obtained with slow infusion of MgSO_4_ to a total dose of 2g. We administered magnesium since it is widely reported in the anesthetic management of pheochromocytoma crisis as a vasodilator. The mechanism of magnesium’s action lies in its ability to decrease catecholamine release. It is also a highly effective α-adrenergic antagonist and anti-arrhythmic drug. Furthermore, magnesium appears to be predominantly an arteriolar dilator, reducing peripheral resistance but with minimal effect on venous return and therefore maintaining cardiac output. The ability of MgSO_4_ to control hemodynamic disturbances in the presence of extremely large catecholamine concentrations was demonstrated by the high levels of cardiovascular control obtained during surgery in our patient’s case [15].

## Conclusions

Undiagnosed functional paragangliomas carry high morbidity and mortality rates and multiple pharmacotherapeutic interventions have been proposed to minimize intra-operative cardiovascular complications. Only a few cases have been reported regarding management of accidental intra-operatively diagnosed functional paragangliomas where anesthesia was conducted via the combination of epidural analgesia and general anesthesia.

This combination of epidural and general anesthesia and the availability of rapid, short-acting vasodilatators (urapidil, MgSO4) and β-blockers (esmolol) led to satisfactory management of a pre-operatively undiagnosed hormonally active tumor in our patient’s case.

It is important to have in mind the possible presence of this usually clinically silent but still hormonally active tumor when sudden unexplained hemodynamic instability occurs intra-operatively. Also, it is important to suspect and appropriately investigate the existence of paragangliomas in every case of retroperitoneal tumor, even in a hemodynamically stable and asymptomatic patient. A thorough differential diagnosis is essential in order to plan the best treatment and therefore gain the best outcome for the patient.

## Consent

Written informed consent was obtained from the patient for publication of this case report and any accompanying images. A copy of the written consent is available for review by the Editor-in-Chief of this journal.

## Competing interests

The authors declare that they have no competing interests.

## Authors’ contributions

BK was chief surgeon and provided the medical documents for the reported case. AS provided the histological examination of the extra-adrenal paraganglioma, supervised the study, and performed critical revision of the manuscript for important intellectual content. KT was principal anesthesiologist and investigator, wrote the manuscript and obtained informed consent. JPS reviewed our patient’s medical records. NVF and IA performed critical revision of the manuscript. All authors contributed to writing the manuscript. All authors read and approved the final manuscript.
